# Assessing Colorectal Cancer Screening Behaviors and Knowledge among At-Risk Hispanics in Southern New Mexico*

**DOI:** 10.4236/jct.2013.46A2003

**Published:** 2013-07

**Authors:** Janeth I. Sanchez, Rebecca Palacios, Beti Thompson, Vanessa Martinez, Mary A. O’Connell

**Affiliations:** 1 Plant and Environmental Sciences, New Mexico State University, Las Cruces, New Mexico, USA; 2 Public Health Sciences, New Mexico State University, Las Cruces, New Mexico, USA; 3 Fred Hutchinson Cancer Research Center, Seattle, Washington, USA

**Keywords:** Colorectal Cancer, Health Disparity, Hispanics, Knowledge, Knowledge, Screening

## Abstract

**Purpose:**

Colorectal cancer (CRC) mortality rates in New Mexico (NM) continue to be higher than national rates. Hispanic CRC mortality rates in NM surpass those of overall Hispanics in the US. This study was designed to characterize and understand factors contributing to low CRC screening rates in this border region.

**Methods:**

A CRC Knowledge Assessment Survey (KAS) was administered in either English or Spanish to 247 individuals attending community events throughout southern NM. A subset of these individuals completed an online CRC risk assessment survey managed by the National Cancer Institute (NCI). Data analysis tested for significant differences in knowledge, physician-patient CRC interactions, CRC risk level perception, and screening rates across diverse ethnic and age groups.

**Results:**

Both CRC knowledge and physician-patient CRC interactions were positively associated with participant screening history. Significant age and ethnic differences for CRC knowledge, physician-patient CRC interactions, and screening history in the NM border sample were also seen. Age-eligible Hispanics (50+) as well as those less than 50 years of age had lower CRC knowledge and were less likely to engage in physician-patient CRC interactions than non-Hispanic Whites (NHWs). The age-eligible Hispanics also reported lower CRC screening rates than their NHW counterparts.

**Conclusions:**

Low CRC knowledge and limited physician-patient CRC interactions appear to contribute to low screening rates in this NM population. Expanding education and outreach efforts for this border population are essential to promote early CRC detection and thereby decrease overall CRC mortality rates.

## 1. Introduction

Colorectal cancer (CRC) is one of the leading causes of death by cancer in the United States (US) with an estimated 143,000 new cases and 52,000 deaths from CRC in 2012 [1]. Recent decades have witnessed a great progress against CRC with overall US CRC mortality rates dropping from 23.7 per 100,000 in 1990 to 17.6 per 100,000 in 2007—a decrease of 25.7% [2, 3].

Despite national trends, decreases in mortality rates have not been equally observed across all 50 US states. For example, mortality rates in New Mexico (NM) decreased only 12% over this same time [2]. New Mexico also shows a unique pattern of incidence and mortality rates. The National Cancer Institute (NCI) estimated that from 2003 to 2007 the CRC incidence and mortality rates for non-Hispanic Whites (NHW) were higher than for Hispanics nationwide (Table 1) [4, 5]. In NM, however, the pattern for mortality rates is reversed with Hispanics showing greater mortality rates than NHWs [6].

Overall, it is thought that mortality rates for CRC have decreased over the years, primarily due to increased CRC screenings [7]. However, there remains a concern that racial/ethnic disparities in screening practices have contributed to NM’s modest declines in CRC mortality. Below we examine factors that may contribute to lower screening and hence higher CRC mortality rates among Hispanics in NM.

### 1.1. Factors Associated with CRC Screening

CRC is a disease that can be prevented by practicing healthy behaviors; in addition, receiving screening tests for CRC at the recommended ages can lead to early detection and eradication of polyps that might become cancerous [8,9]. The Centers for Disease Control and Prevention (CDC), and the U.S. Preventive Services Task Force (USPSTF) recommend individuals begin colorectal cancer screening with the use of a fecal occult blood test (FOBT), sigmoidoscopy, or colonoscopy at age 50 if there is no family history of CRC [10,11]. Recent research, however, suggests that physicians should begin discussing CRC among average-risk individuals at age 40 [12–14]. Individuals at average risk are asymptomatic and have no genetic risk factors for CRC, including having a personal or family history of CRC, adenomatous polyps, or Crohn’s disease [15]. However, in the US only about 50% of Americans 50 years old or older receive routine screening^1^ [16–18]. Healthy People 2020 now targets a nationwide CRC screening rate of 70.5% of all age-eligible adults [18].

In 2010, the CDC used data from the Behavioral Risk Factor Surveillance System (BRFSS) to evaluate routine CRC screening rates in the US; NM was among a group of states exhibiting the lowest levels of routine CRC screening (54.1% to 59.2%) [19]. Consistent with these data, the NM Department of Health estimated that 55% of the 50 years and older population had received routine CRC screening [20]. These rates are lower for the state’s Hispanic population where the BRFSS surveys show only 45.7% of Hispanics had received CRC screening in the state [21]. The low CRC screening rates in NM, particularly among Hispanics residents, could be attributed to a variety of factors including socioeconomic status (SES), lack of CRC knowledge, and lack of physiccian-patient CRC interactions.

### 1.2. SES Barriers to Screening

There are several socioeconomic factors that appear to be associated with low screening rates in NM. Border Hispanics, for example, experience high rates of poverty and low rates of health insurance. In addition, the median household income in NM between 2007 and 2011 was $44,631, approximately $8000 less than the national level [22]. A high proportion (18.4%) of NM residents live below poverty level compared to 13.8% of the national population. Poverty rates are even higher in NM border counties, Doña Ana, Luna, and Hidalgo (24.5%, 32.8%, and 22.6%, respectively) [23,24]. Income and poverty levels could be associated with lack of health insurance coverage for screening tests. In NM the rate of no health insurance coverage is higher than the national average, 21% vs 15.9% [25,26]. Hispanics and Native Americans in NM lack health insurance at higher rates compared to NHWs (23.0%, 28.3%, and 10.9%, respectively) [26].

### 1.3. Knowledge Barriers to CRC Screening

Lack of knowledge of risk factors and personal risk may be a significant barrier to screening [27,28]. Sanderson and colleagues [29] assessed CRC knowledge and screening rates among underserved populations; respondents with little or no formal schooling had lower knowledge of CRC and lower CRC screening rates than participants with higher educational levels. The lack of screening practices among minority populations may be due to the lack of CRC knowledge and access to reliable, current, and culturally appropriate information on CRC risk factors (diet, exercise, screening, and smoking) and screening modalities [2,30–32]. For example, Akhtar and colleagues [33] found that among first-degree relatives with CRC, only 59% had knowledge of their own increased individual risk. In addition, only about half of first-degree relatives had knowledge of modifiable risk factors for CRC (e.g., diet, exercise, and alcohol consumption). The majority of these participants (94%) felt that they were not informed of their increased risk due to a CRC family history and 88% of the participants stated they would change their lifestyle if enough information was given to them [33].

### 1.4. Physician-Patient Interactions Regarding CRC Screening

Screening recommendations by physicians also contribute to CRC screening rates among at-risk individuals [14,34]. Using physician and population-based surveys in NM, Hoffman and colleagues [12] identified differences in perceived barriers to CRC screening between physiccians and patients. Specifically, they found that adults 50 and older living in northern NM reported that a lack of physician recommendation and lack of CRC symptoms were barriers to CRC screening. Physicians, in contrast, reported that a lack of screening knowledge served as the major barrier to CRC screening among their patients [12]. Although patients and physicians in this study correctly identified contributing factors to low CRC screening, they clearly had different perspectives on the main contributors.

### 1.5. Present Study

Southwestern NM residents participated in an assessment of CRC knowledge, past physician-patient interactions regarding CRC screening, CRC screening history, and behavioral intentions to obtain CRC screening in the future. Finally, this study assessed participant CRC risk using the NCI’s online CRC Risk Assessment Tool and compared their rates to national risk rates. Overall this study was designed to characterize and understand factors contributing to low CRC screening rates in this border region.

## 2. Methods

Data for this study was gathered at 17 community health events in nine NM border cities. This study was conducted between March 2011 and March 2012. The New Mexico State University Institutional Review Board (FWA00000451) approved all study procedures and the survey instruments (NMSU IRB approval #441).

### 2.1. Participants and Recruitment

This study used a convenience sample of 247 participants who attended a CRC educational booth at one of the 17 community events that were conducted in the NM border region. A subset of the participants (n = 90) completed the NCI’s free online CRC Risk Assessment Tool [35] and consented to the use of their results as part of this study. Participants were recruited by placing flyers at various locations, including public libraries and municipal senior centers, in the targeted regions. Information about the CRC education booth was also distributed using a weekly radio station show that broadcasts in all of southern NM and features health related news and events. Regional organizers of health fairs and community events also promoted the CRC education booth in their cities.

### 2.2. Measures and Instruments

#### 2.2.1. The CRC Knowledge Assessment Survey (KAS)

The CRC KAS consists of 25-items developed based on CRC risk information from the NCI, as well as on literature using health surveys in underserved populations [29–32,36,37]. This survey assessed CRC knowledge (14 items), CRC screening history (3 items), behavioral intentions to get screened (2 items), and physician-patient interactions regarding CRC (2 items). The three knowledge subcategories included general CRC knowledge (2 items), CRC screening knowledge (7 items) and CRC risk factor knowledge (5 items). The Simple Measure of Gobbledygook (SMOG), a readability assessment, was utilized to evaluate the literacy levels of the survey and the consent forms. The readability grade level for the KAS was estimated to be 7.9. The CRC KAS was developed in English, translated to Spanish, and back translated to English by a native bilingual speaker with degrees in Spanish and Health Sciences.

#### 2.2.2. NCI’s Online CRC Risk Assessment Tool

This assessment consists of 19 items for women and 23 items for men and calculates the risk of developing CRC among individuals aged 50 years or older [38,39]. This tool takes approximately 5 to 8 minutes to complete and assesses demographics including ethnicity, age, sex, height, and weight. The tool also measures various CRC risk factors, such as fruit and vegetable consumption, physical activity, nonsteroidal anti-inflammatory drug use, CRC family history, and CRC screening history. The tool assesses gender specific risk factors including smoking for males and menopause onset and use of hormone replacement therapy for females. Overall, the assessment tool provides an estimated risk of developing CRC compared to the national average of individuals of the same age, gender, and ethnic group as the respondent. The 5-year-risk is consistent with 10 year and lifetime risk, so in order to reduce redundancy in outcomes, the 5-year-risk was incorporated as the sole dependent variable from this assessment.

The NCI’s Online CRC Risk Assessment Tool outcomes were recorded in an Excel database. This tool is not currently available in Spanish; however, project staff assisted participants who only spoke Spanish to complete the online assessment. Native bilingual staff directly translated the questions on the tool and then back-translated the participants’ answers to English and entered the response in the tool. This tool should be used with the 50 and older age group, however, individuals younger than 50 years who wanted to assess their risk for CRC were informed that their risk estimate would not be as accurate as those who were at least 50 years old. Given that this tool may assist patients and their health care providers in making informed decisions about when to begin CRC screening, all participants completing this assessment were given a copy of their results and were strongly advised to consult with a physician regarding their CRC risk.

### 2.3. Procedures

Health fair participants visiting the CRC booth were asked to complete the KAS and the NCI’s CRC Risk Assessment Tool. The KAS was available for completion using paper and pen, while laptops and a wireless connection were set up at the CRC educational booths for individuals to complete the Risk Assessment Tool. The project staff made a concerted effort to encourage those who were at least 50 years old to participate in the assessment, although all attendees to the booth were invited to complete the surveys. Written informed consent from these individuals was obtained prior to their participation. The survey and consent forms were available in English and Spanish; staff members conducting the surveys were bilingual in English and Spanish. Compensation for a participant’s time was given in the form of a tote bag that contained a planner and NCI CRC information in either English or Spanish.

### 2.4. Data Analysis

Data analysis was performed using SPSS: Version 19.0. Logistic regression analyses were used to examine the effects of participant knowledge (categorical variable) and past physician recommendations on screening history. Two way MANOVAs were conducted using ethnicity and age group as the independent variables and all CRC related variables (e.g., CRC knowledge, CRC physician interactions, CRC screening history, behavioral intentions to obtain screening, and CRC risk level) as the dependent variables. Correlational analyses were conducted to explore factors most closely associated with past participation in CRC screening.

#### 2.4.1. Data Reduction

Composite variables were created for conceptually related variables (Table 2). Scales and subscales demonstrating reasonable internal reliability were converted into composite scores consisting of sum scores, as described below.

#### 2.4.2. Knowledge

A total knowledge composite score consisting of all fourteen knowledge items was computed (α = 0.942). Composite scores were also calculated for the three knowledge subscales: General CRC knowledge (2 items; α = 0.740), Screening knowledge (7 items; α = 0.893), and CRC risk factor knowledge (5 items; α = 0.877). For logistic regression, total knowledge was converted to a categorical variable with three levels. Knowledge scores of three or lower were considered low knowledge, scores of four to nine reflected moderate knowledge, and scores of ten or greater reflected high knowledge.

#### 2.4.3. Past Physician-Patient Interactions Regarding CRC

A composite variable consisting of the sum of two variables assessing past CRC-related interactions with one’s physician was calculated (2 items; α = 0.916).

## 3. Results

### 3.1. Participants

The sample recruited from the health fairs and community events to complete the KAS was predominantly Hispanic female; details are presented in Table 3. Slightly more than half of the participants (54.7%) were at least 50 years old and 16.2% were between the ages of 40 and 49. When asked about their ethnic/racial classification, 62.8% self-reported as Hispanic, with 87% of these being Mexican American or of Mexican descent; 35.6% selfreported as non-Hispanic White; and 1.6% as other (e.g., Native American/American Indian). Demographics for the study sample largely reflect demographics for the NM border region. A subset of the participants (90 individuals) completed the NCI’s online CRC Risk Assessment Tool. The ethnicity of this subset was: 44 Hispanics, 44 NHW and 2 Native American/American Indians.

### 3.2. Logistic Regression

We examined the effects of participant knowledge and physician recommendations to get screened on CRC screening history using logistic regression analyses. Results of these analyses showed that both doctor recommendations and knowledge level contributed to screening history. Physicians’ recommendations had a significant effect (B = 3.65; p < 0.001) on increasing one’s chances of being screened for CRC by 38.5 times (CI: 11.9 to 124.6). Knowledge also contributed independently to screening history. Using “low” knowledge as the reference category, simple contrasts revealed that high knowledge (B = 2.38; p < 0.01) but not moderate knowledge (B = 0.85, n.s.) significantly increased one’s chances of being screened for CRC. Individuals with high knowledge were 10.8 (CI: 2.5 to 47.1) times more likely to be screened than those having low knowledge. Although not significant, individuals with moderate knowledge were 2.3 (CI: 0.50 to 10.9) times more likely to be screened than individuals with low knowledge.

We were unable to examine the effects of participant knowledge and physician recommendations on behaveioral intentions to get screened in the future due to a lack of variance in this dependent variable. Among this subsample, the majority (97%) reported they intended to be screened for CRC in the future. This dependent variable was eliminated from all subsequent analyses and tables.

### 3.3. CRC Knowledge

In order to examine knowledge differences across ethnic and age groups, a two-way MANOVA with ethnicity and age-group as the between-subjects factors was conducted using the knowledge composite variables (general CRC knowledge, screening knowledge, CRC risk factor knowledge) as the dependent variables. The results of this analysis revealed significant multivariate effects for both ethnicity, Wilks’ Λ = 0.90, F(3, 218) = 8.75, p < 0.001, and age-group, Wilks’ Λ = 0.96, F(3, 218) = 3.13, p < 0.05. No interaction was found.

Univariate ANOVAs revealed significant ethnicity effects for general CRC knowledge, screening knowledge, and CRC risk factor knowledge (Table 4). Hispanics reported lower scores on all three-knowledge categories compared to NHWs. Univariate ANOVAs for age were significant for all three knowledge subcategories. Individuals younger than 50 scored lower in all three knowledge categories compared to those older than 50 years of age.

### 3.4. Physician-Patient Interactions Regarding CRC

A univariate ANOVA was conducted with ethnicity and age-group as the between-subjects factors and physician interactions composite variable as the dependent variable. These analyses revealed significant ethnicity and age group effects for past physician-patient interactions (Table 4). Hispanics reported significantly fewer past physician-patient interactions regarding CRC compared to NHWs. Individuals younger than 50 reported fewer past physician-patient interactions regarding CRC compared to the 50 years and older group. The interaction was not significant.

### 3.5. CRC Screening History

A univariate ANOVA was conducted with ethnicity and age-group as the between-subjects factors and CRC screening history as the dependent variable. These analyses revealed significant ethnicity and age group effects for CRC screening history (Table 4). Hispanics reported significantly lower past CRC screening compared to NHWs. Individuals younger than 50 also reported lower past CRC screening compared to the 50 years and older group. The interaction was not significant.

Although the sample size for the 40 to 49 age group was small (n = 40), a univariate ANOVA was conducted with ethnicity as the between subjects factor and CRC screening history as the dependent variable in this specific age group. This test revealed a significant ethnicity effect with Hispanics reporting lower CRC screening than NHWs in the 40 to 49 age group. Table 4 shows that where as 50% of NHWs in this age group had already received a colonoscopy only 8% of Hispanics had received such CRC screening.

### 3.6. Behavioral Intentions to Discuss CRC with a Physician

A univariate ANOVA was conducted with ethnicity and age-group as the between-subjects factors and behavioral intentions to discuss CRC with physician as the dependent variable. These analyses revealed significant age group effects for behavioral intentions to discuss CRC with a physician (Table 4). Individuals 50 years and older reported greater intentions to discuss CRC with a physician compared to individuals younger than 50. Tests for ethnicity and the interaction were not significant.

### 3.7. NCI’s CRC Risk Assessment Tool

**Figure 1:**
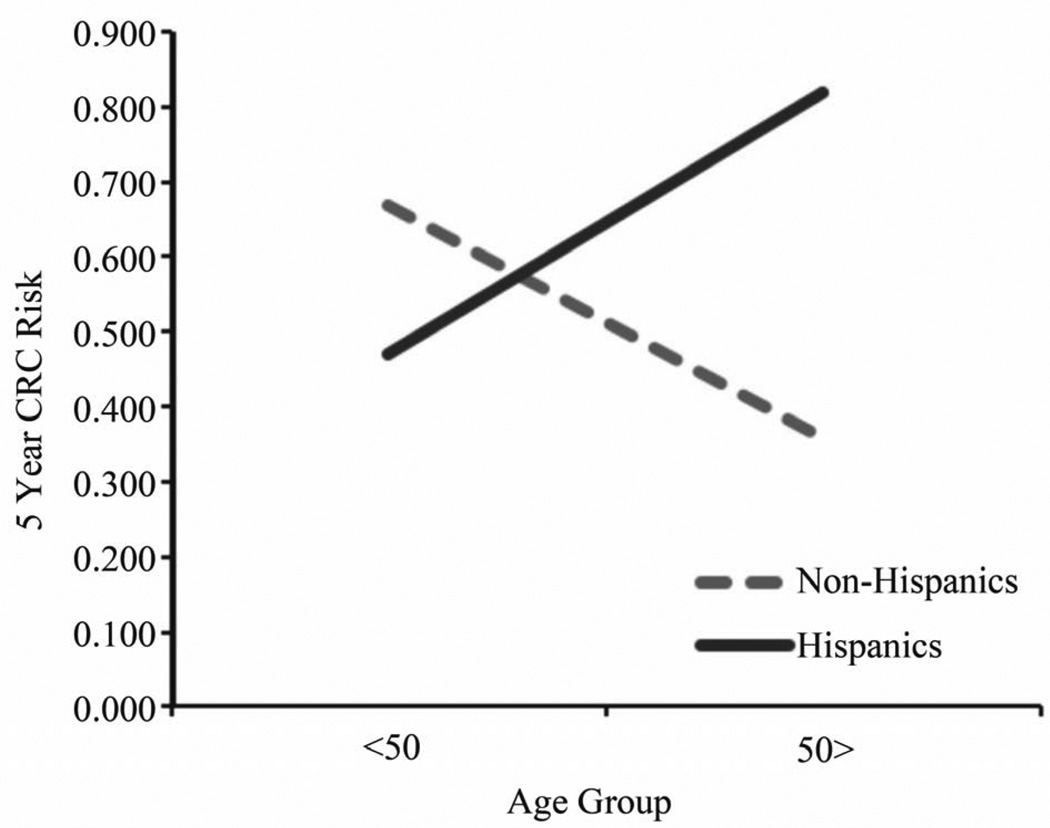
National cancer institute CRC risk assessment by age and ethnicity.

A univariate ANOVA was conducted with ethnicity and age-group as the between-subjects factors and 5 year CRC risk as the dependent variable. This analysis revealed no significant main effects, however, there was a significant interaction, F (1, 84) = 8.26, p < 0.01 (Figure 1). Older (>50) Hispanics demonstrated greater 5-year CRC risk compared to younger Hispanics, F (1, 45) = 11.57, p < 0.01. Although the trend was reversed for NHWs, the age group effect was not significant.

### 3.8. Correlations

We performed zero-order correlations to investigate the relationship of CRC knowledge and past physician-patient CRC interactions to CRC screening history and behavioral intentions (Table 5). All knowledge categories and physician interactions positively correlated with CRC screening history and behavioral intentions to discuss CRC with physician.

## 4. Discussion

Colorectal cancer is a disease that can be prevented and successfully treated through early detection using screening tests, education, and changes in lifestyle behaviors [40,41]. In this study we examined the effects of CRC knowledge and CRC physician-patient interactions on CRC screening behaviors and intentions across ethnic and age groups. In addition, CRC risk was also examined across ethnic and age groups. CRC continues to be a concern as the screening rates are still low among the population as a whole and are especially low among certain ethnic groups and in certain regions of the US [7,20]. By identifying barriers to CRC screening among border Hispanics, interventions may be better tailored to effecttively promote CRC screening and engage this population in timely treatment for CRC.

### 4.1. Predictors of CRC Screening

This study found that both participant CRC knowledge and past physician-patient CRC interactions were strongly and independently related to CRC screening history. Indeed, high levels of CRC knowledge significantly increased the odds of having been screened for CRC among New Mexicans. Moderate CRC knowledge, in contrast, also increased one’s odds of having experienced CRC screening over those with low CRC knowledge, but to a lesser extent. Our findings are consistent with those of Arnold *et al*. [42] who reported high CRC literacy levels are strong predictors of CRC screening.

In addition to screening history, this study also examined participants’ behavioral intentions to discuss CRC with their physician, and to participate in CRC screening in the future. Both CRC knowledge and past physiccian-patient CRC interactions were positively correlated to future intentions to discuss CRC with a physician. In contrast, neither CRC knowledge nor past physician-patient CRC interactions related to future intentions to participate in CRC screening. This outcome may be explained by the lack of variation in intentions to get screened for CRC. Specifically, almost all individuals who were age-appropriate for CRC screening, but who had never been screened for CRC, reported that they intended to be screened for CRC in the future.

### 4.2. Ethnic Effects

Hispanics in this study scored lower on all CRC knowledge subcategories (general, screening and risk) compared to NHWs. These findings are important because past research shows a positive relationship between CRC knowledge and screening behaviors in minority populations [1,8,12,16]. Furthermore, increased knowledge of CRC risk factors can aid individuals in making informed decisions to modify those risk factors and engage in CRC screening [27,28,33]. Future interventions should focus on increasing knowledge of CRC, personal risk for developing CRC and screening modalities and locations among the border Hispanic population.

In addition to lower CRC knowledge, Hispanics in this study also reported fewer CRC interactions with their physician, including discussing CRC and the importance of being screened for CRC at the appropriate ages. Our results identify a key barrier for Hispanics. Hoffman *et al*. [12] found that the lack of CRC interactions with physiccians served as a barrier to CRC screening in New Mexicans. Healthy People 2020 also identify physician CRC interactions as the single most important predictor of CRC screening in patients [18]. Therefore, encourageing physicians to discuss CRC with their patients-particularly those in the recommended screening ages and minority groups with high risk should be a key component of physician training. Such training is particularly important as physicians demonstrate a tendency to believe that patient knowledge is more important to increasing CRC screening than physicians’ recommendations [12].

Given the low levels of CRC knowledge and reduced physician-patient CRC interaction found among Hispanics, it is not surprising that these individuals also reported lower past CRC screening compared to NHWs. These findings support the need for improving Hispanic health literacy for CRC and promoting physician discussions/recommendations for CRC with Hispanic patients.

Importantly, no ethnic differences were found in behavioral intentions to engage in CRC discussions with physicians. Overall, 93.4% reported that they intended to discuss CRC with their physicians.

### 4.3. Age Differences

As might be expected, individuals at least 50 years of age exhibited higher levels of knowledge on all subcategories of CRC knowledge. However, since screening guidelines recommend that individuals identified as “high risk” be screened as early as 40 years [12–14], middle aged adults should begin acquiring CRC knowledge earlier in life. These recommendations for early education and screening may be particularly relevant to border Hispanics who demonstrate higher levels of poor nutrition, physical inactivity, and other modifiable risk behaviors for CRC [43,44].

Participants younger than age 50 reported fewer CRC interactions with their physician. This pattern was expected given that national recommendations for CRC screening target individuals 50 years of age and older [10]. As above, however, physician education might emphasize the importance of targeting individuals younger than 50 years who have a family history of CRC and/or who exhibit high risk for developing CRC. Specifically, physicians should be aware of recommendations to initiate CRC discussions with their average-risk patients who are at least 40 years old [13,14,29].

Consistent with national guidelines recommending that CRC screening begin at 50 years [10], this study found that individuals 50 years and older reported greater past CRC screening compared to the less than 50 age group. However, overall CRC screening rates for the border population of screening age were somewhat lower (51%) than screening rates found at the state and national level [13,14,16,17,20,22,45]. Furthermore, Hispanics in this study reported significantly lower CRC screening rates than NHWs (37.5% and 70%, respectively). This same pattern for ethnicity was identified among the 40 to 49 age group, where only 8% of Hispanics in this age group had received CRC screening compared with 50% of the NHWs.

This study also identified age group differences in behavioral intentions to discuss CRC with a physician. Specifically, the 50 plus age group demonstrated greater intentions to engage in CRC related interactions with a physician than their younger counterparts. We found this age-based difference for both Hispanic and NHW groups. Overall, study participants demonstrated high behavioral intentions to discuss CRC with their physician and to obtain CRC screening in the future. High poverty and low rates of health insurance coverage in NM may serve as barriers to CRC screening, particularly among border Hispanics. Interventions that promote increasing healthcare coverage and access to low or reduced cost screening services may benefit individuals with high behavioral intentions to participate in CRC screening but no financial means for doing so. Furthermore, ensuring the public is aware of screening locations in their community may also facilitate converting high behavioral intentions to obtain screening to actual high screening rates.

### 4.4. CRC Risk Assessment

This study identified an interesting interaction using the NCI’s CRC Risk Assessment Tool. As one might expect, CRC risk should be greater in the 50 years and older age group compared to the less than 50 years age group. This age-related increased risk pattern was identified in Hispanics but not in NHWs. NHWs demonstrated a trend toward greater 5 year CRC risk in the less than 50 years age group relative to the 50 years and older age group, although this effect was not statistically significant. This lower risk in later age identified among NHWs may be explained by the relative differences in CRC awareness and knowledge between Hispanics and NHWs. Specifically, knowledge attainment of CRC risk factors throughout the lifetime of NHWs may lead them to reduce high-risk behaviors for CRC (e.g., poor nutritional practices, physical inactivity and smoking) as they get older. Hispanics, in contrast, may not be acquiring such CRC knowledge in their lifetime and therefore may be less equipped to reduce their CRC risk at a later age. The low screening rates among Hispanics compared to NHWs will also increase the risk score for this group.

### 4.5. Limitations

Predictors of CRC screening in this study were limited to CRC knowledge and physician-patient CRC interactions. Although we acknowledge that knowledge on CRC by itself is insufficient to promote CRC screening behaviors, Beydoun & Beydoun [34] suggest that increasing knowledge is an essential component of any intervention whose ultimate goal is to reach target cancer screening rates as recommended by national health organizations. We not only identified knowledge as an important correlate of CRC screening history, but distinguished between knowledge levels necessary to actually promote CRC screening behaviors. High levels of CRC knowledge are necessary to increase the odds of a person participating in screening.

A variety of other factors may also have contributed to low CRC screening practices in the targeted border population. For example, SES, which was not assessed in this study, may be one such factor. Individuals of low socioeconomic status are less likely to have health care coverage or access to a regular provider [46]. The NM border region has high poverty and low education levels, particularly among Hispanic residents [21,31,36,47].

The self-report methodology used in this study is an additional limitation. Verification of CRC screening through medical records would have been more accurate in estimating actual screening rates in this population, however, that was not possible. Finally, another limitation of this study was the modest sample size, particularly regarding the subsample that completed the NCI’s CRC Risk Assessment Tool.

## 5. Conclusions

This study expands on existing knowledge regarding CRC, particularly as it relates to the unique NM border population, a population not targeted in previous research. The first key finding of this study was the relationship of CRC knowledge level to CRC screening history. This is particularly relevant for the Hispanic border population, which demonstrated significantly lower CRC knowledge levels when compared to NHWs residing in this same region. Intervention programs designed to increase CRC screening in the border region should significantly enhance CRC awareness and knowledge, and do so in a culturally appropriate manner. These educational efforts should be coupled with mechanisms that facilitate access to healthcare (e.g., information on screening locations and low cost or free cancer programs; applications for healthcare coverage), promote tools and skills to advocate for their health with their physicians (e.g., discussion topics and sample questions for physicians) and address psychosocial factors (e.g., negative perceptions about CRC screening methods and outcomes; screening tests effectiveness) [34,48].

Although physician recommendations have been clearly linked to CRC screening history in previous research [12,14,34], a second finding of our study was the substantial healthcare disparities for border Hispanics. As noted, border Hispanics are simply not receiving recommendations for CRC screening. This may be attributed to lack of a regular provider or to a lack of recommendation from one’s primary care physician. In this regard, the border Hispanic population would benefit from state and national programs designed to increase 1) health care coverage; and 2) physician and resident training programs emphasizing the importance of promoting CRC screening among their racial/ethnic minority patients.

A third finding of this study was that ethnicity moderated the 5 year risk identified for different age groups. Although young Hispanics (*i.e*., less than 50 years) demonstrated a lower risk for CRC than did young NHWs, older Hispanics (*i.e*., 50 plus) demonstrated a significantly higher CRC risk compared to their NHW counterparts. This finding suggests border Hispanics would benefit from early CRC education in order to promote healthier lifestyle practices starting in young adulthood with the ultimate goal of reducing lifetime risk for CRC.

Our results suggest that at-risk minority populations, such as those found in the NM border region, would benefit from early educational interventions for CRC, interventions designed to significantly increase CRC knowledge. Such educational interventions should emphasize characteristics of the disease and its progression, related risk factors, screening modalities and community locations for screening services. Furthermore, CRC educational interventions should increase patient capacity to discuss CRC with their physicians. Expanding education and outreach efforts for this border population is essential to promote early CRC detection and decrease overall CRC mortality rates.

## Figures and Tables

**Table 1:** CRC rates in Hispanic and NHW groups.

Region	Incidence (per 100,000)		Mortality (per 100,000)	

	*NHW*	*Hispanic*	*NHW*	*Hispanic*
USA [5]	49.0	39.3	17.5	12.9
NM [6]	45.1	41.8	14.8	17.4

**Table 2:** CRC knowledge assessment survey: items and scale internal reliabilities.

Scale	Items	Cronbach’s Alpha
Total knowledge	All 14 knowledge items	0.94
General knowledge of CRC	Do you know what cancer of the colon and rectum (CRC) is?	0.74
	Do you know what a colon polyp is?	
	A low fat and high fiber diet helps decrease the risk for cancer of the colon and rectum.	
	Physical activity decreases the risk for cancer of the colon and rectum.	
Knowledge of CRC risk factors	The risk of colon and rectum cancer increases after the age of 50.	0.88
	A family history of cancer of the colon and rectum does not increase your risk.	
	Do you know what your risk for colorectal cancer is?	
	Finding cancer early will not increase the chances of surviving it.	
	You only need to have a colorectal cancer screening test if you are having symptoms.	
	Do you know the different types of screenings for cancer of the colon and rectum?	
Knowledge of screening	Do you know what a:	0.89
	Fecal Occult Blood Test (FOBT) is?	
	Colonoscopy is?	
	Sigmoidoscopy is?	
	Do you know where you can receive these screening services?	
Physician interactions	Have you ever talked to your physician about cancer of the colon and rectum?	0.92
	Has your physician ever recommended a FOBT, sigmoidoscopy, or colonoscopy?	

**Table 3:** Participant characteristics.

Characteristic	Number	Percent
Age		
20 – 39	72	29.1
40 – 49	40	16.2
50+	135	54.7
Gender		
Female	188	76.1
Male	59	23.9
Race/Ethnicity		
White (non-Hispanic)	88	35.6
Hispanic	155	62.8
Other	4	1.6

**Table 4:** Knowledge differences across ethnic and age groups.

	Ethnicity					Age Group				

	NHW		Hispanic		F-value	<50 yrs		≥50 yrs		F-value
	
	M	SE	M	SE		M	SE	M	SE	
Knowledge										
Total knowledge	9.07	0.54	5.77	0.39	24.35***	6.45	0.51	8.39	0.43	8.42**
General CRC knowledge	1.44	0.10	1.00	0.07	14.20***	1.05	0.09	1.39	0.08	8.35**
CRC screening knowledge	4.17	0.28	2.43	0.20	26.34***	2.83	0.26	3.77	0.22	7.67**
CRC risk factor knowledge	3.47	0.22	2.34	0.16	17.39***	2.57	0.21	3.24	0.17	6.12*
Physician-patient CRC Interactions										
Physician-patient CRC interactions	1.10	0.09	0.57	0.06	23.08***	0.36	0.08	1.31	0.07	75.40***
CRC Screening History										
Received colonoscopy in the past	0.45	0.05	0.21	0.03	18.28***	0.12	0.04	0.54	0.04	51.94***
Participants aged 40 – 49 years who received colonoscopy in the past	0.50	0.11	0.08	0.04	10.34**	--	--	--	--	--
Behavioral Intentions Regarding CRC										
Plan on discussing CRC with physician in the future	0.93	0.03	0.93	0.02	0.01	0.89	0.03	0.97	0.02	4.1*
NCI Risk Assessment Tool										
Personal 5 year risk over the national average	0.57	0.09	0.59	0.07	0.03	0.51	0.10	0.64	0.06	1.32

* p < 0.05,

** p < 0.01,

*** p < 0.001,

**Table 5:** Relationship of CRC knowledge and physician interacttions with CRC screening history and behavioral intentions.

	Screening history	Intentions to discuss CRC with physician
Total knowledge score	0.64***	0.26***
General CRC knowledge	0.54***	0.31***
CRC risk factor knowledge	0.54***	0.23***
CRC screening knowledge	0.67***	0.22***
Patient-physician CRC interactions	0.78***	0.17***

* p < 0.05,

** p < 0.01,

*** p < 0.001.

## Footnotes

1 Routine screening is defined as obtaining any of the following tests beginning at 50 years old: 1) fecal occult blood test (FOBT) annually; 2) FOBT and flexible sigmoidoscopy every 5 years; 3) double contrast barium enema every 5 years; 4) colonoscopy every 10 years.

## References

[1] American Cancer Society (2012). Cancer Facts and Figures 2012. http://www.cancer.org.

[2] Naishadham D, Lansdorp-Vogelaar I, Siegel R, Cokkinides V, Jemal A (2011). State Disparities in Colorectal Cancer Mortality Patterns in the United States. Cancer Epidemiol Biomarkers Prevention.

[3] Richardson L, Tai E, Rim S, Joseph D, Plescia M (2011). Vital Signs: Colorectal Cancer Screening, Incidence, and Mortality-United States, 2002–2010. Morbidity and Mortality Weekly Report.

[4] Howlander N, Noone A, Krapcho M (2012). SEER Cancer Statistics Review, 1975–2009. http://seer.cancer.gov/csr/1975_2009_pops09.

[5] National Cancer Institute (2013). Surveillance Epidemiology and End Results, Cancer Statistics, Fast Stats, Race and Ethnicity, 2003–2007 Average. Department of Health and Human Services.

[6] New Mexico Tumor Registry (2012). Cancer of the Colon and Rectum in New Mexico. University of New Mexico.

[7] Shapiro JA, Klabunde CN, Thompson TD, Nadel MR, Seeff LC, White A (2012). Patterns of Colorectal Cancer Test Use, Including CT Colonography, in the 2010 National Health Interview Survey. Cancer Epidemiology, Biomarkers & Prevention.

[8] Vernon SW (1997). Participation in Colorectal Cancer Screening: A Review. Journal of the National Cancer Institute.

[9] Maciosek M, Solberg L, Coffield A, Edwards N, Goodman M (2006). Colorectal Cancer Screening: Health Impact and Cost Effectiveness. American Journal of Preventive Medicine.

[10] US Preventative Services Task Force (2008). Screening for Colorectal Cancer: US Preventive Services Task Force Recommendation Statement. Annals of Internal Medicine.

[11] Centers for Disease Control (CDC) (2012). Colorectal Cancer. Department of Health and Human Services.

[12] Hoffman RM, Rhyne R, Helitzer D (2011). Barriers to Colorectal Cancer Screening: Physician and General Population Perspectives, New Mexico, 2006. Preventing Chronic Disease.

[13] Winawer S, Fletcher R, Rex D (2003). Colorectal Cancer Screening And Surveillance: Clinical Guidelines and Rationale—Update Based on New Evidence. Gastroenterology.

[14] Klabunde CN, Lanier D, Nadel MR, McLeod C, Yuan G, Vernon SW (2009). Colorectal Cancer Screening by Primary Care Physicians: Recommendations and Practices, 2006–2007. American Journal of Preventive Medicine.

[15] Read T, Kodner I (1999). Colorectal Cancer: Risk Factors and Recommendations for Early Detection. American Family Physician.

[16] Smith R, Cokkinides V, Eyre H, Society AC (2004). American Cancer Society Guidelines for the Early Detection of Cancer, 2004. CA: A Cancer Journal for Clinicians.

[17] Le H, Ziogas A, Lipkin SM, Zell JA (2008). Effects of Socioeconomic Status and Treatment Disparities in Colorectal Cancer Survival. Cancer Epidemiology, Biomarkers & Prevention.

[18] Office of Disease Prevention and Health Promotion (2012). Healthy People 2020: Improving the Health of Americans. Department of Health and Human Services.

[19] Centers for Disease Control (CDC) (2010). Behavioral Risk Factor Surveillance System Survey Data. http://www.cdc.gov/cancer/colorec-tal/statistics/screening_rates.htm.

[20] (2007). New Mexico Department of Health,"New Mexico Cancer Facts and Figures. http://www.cancernm.org/cancercouncil/facts_figures.htm.

[21] Gonzales M, Nelson H, Rhyne RL, Stone SN, Hoffman RM (2012). Surveillance of Colorectal Cancer Screening in New Mexico Hispanics and Non-Hispanic Whites. Journal of Community Health.

[22] US Census Bureau (2012). State and County QuickFacts: New Mexico. http://quickfacts.census.gov/qfd/states/35000.html.

[23] US Census Bureau Small Area Income and Poverty Estimates: State and Country Maps. http://www.census.gov/did/www/saipe/data/statecounty/maps/2010.html.

[24] US HUD (2008). US Department of Housing and Urban Development: Homes & Communities. Facts about Farm Workers and Colonias. http://www.hud.gov/groups/farmwkercolonia.cfm.

[25] US Census Bureau (2012). Health Insurance Coverage Status. 2009–2011 American Community Survey 3-Year Estimates. http://factfinder2.census.gov/faces/tableservices/jsf/pages/productview.xhtml?pid=ACS_11_3YR_S2701&prodType=table.

[26] New Mexico Human Services Department (2008). New Mexico Uninsured Population and Income Data. http://www.hsd.state.nm.us/pdf/UninsuredPopulationData5-14-08.pdf.

[27] Kim S, Perez-Stable E, Wong S (2008). Association between Cancer Risk Perception and Screening Behavior among Diverse Women. Archives of Internal Medicine.

[28] Shin DW, Kim YW, Oh JH (2011). Knowledge, Attitudes, Risk Perception, and Cancer Screening Behaviors among Cancer Survivors. Cancer.

[29] Sanderson P, Weinstein N, Teufel-Shone N, Martinez M (2011). Assessing Colorectal Cancer Screening Knowl edge at Tribal Fairs. Preventing Chronic Disease.

[30] Wolf M, Rademaker A, Bennet C (2005). Development of a Brief Survey on Colon Cancer Screening Knowledge and Attitudes among Veterans. Preventing Chronic Disease.

[31] Coronado GD, Golovaty I, Longton G, Levy L, Jimenez R (2011). Effectiveness of a Clinic-Based Colorectal Cancer Screening Promotion Program for Underserved Hispanics. Cancer.

[32] Dolan NC, Ferreira MR, Davis TC (2004). Colorectal Cancer Screening Knowledge, Attitudes, and Beliefs among Veterans: Does Literacy Make a Difference?”. Journal of Clinical Oncology.

[33] Akhtar S, Sinha S, McKenzie S, Sagar PM, Finan PJ, Burke D (2008). Awareness of Risk Factors Amongst First Degree Relative Patients with Colorectal Cancer. Colorectal Disease.

[34] Beydoun HA, Beydoun MA (2008). Predictors of Colorectal Cancer Screening Behaviors among Average-Risk Older Adults in the United States. Cancer Causes & Control.

[35] National Cancer Institute (2009). Colorectal Cancer Risk Assessment Tool. National Institutes of Health.

[36] Fernandez ME, Wippold R, Torres-Vigil I (2008). Colorectal Cancer Screening among Latinos from U.S. Cities along the Texas-Mexico Border. Cancer Causes & Control.

[37] Johnson-Kozlow M (2010). Colorectal Cancer Screening of Californian Adults of Mexican Origin as a Function of Acculturation. Journal of Immigrant and Minority Health.

[38] Freedman AN, Slattery ML, Ballard-Barbash R (2009). Colorectal Cancer Risk Prediction Tool for White Men and Women without Known Susceptibility. Journal of Clinical Oncology.

[39] Park Y, Freedman AN, Gail MH (2009). Validation of a Colorectal Cancer Risk Prediction Model among White Patients Age 50 Years and Older. Journal of Clinical Oncology.

[40] Coups EJ, Manne SL, Meropol NJ, Weinberg DS (2007). Multiple Behavioral Risk Factors for Colorectal Cancer and Colorectal Cancer Screening Status. Cancer Epidemiology Biomarkers and Prevention.

[41] Huxley RR, Ansary-Moghaddam A, Clifton P, Czernichow S, Parr CL, Woodward M (2009). The Impact of Dietary and Lifestyle Risk Factors on Risk of Colorectal Cancer: A Quantitative Overview of the Epidemiological Evidence. International Journal of Cancer.

[42] Arnold CL, Rademaker A, Bailey SC (2012). Literacy Barriers to Colorectal Cancer Screening in Community Clinics. Journal of Health Communication: International Perspectives.

[43] Palacios R, Kittleson M, Rodriguez-Herrera J (2012). Obesity in the 51st State. Health Education Monographs.

[44] Hlaing W, Nath SD, Huffman FG (2007). Assessing Overweight and Cardiovascular Risks among College Students. American Journal of Health Education.

[45] American Cancer Society (2012). Colorectal Cancer Early Detection. http://www.cancer.org/acs/groups/cid/documents/webcontent/003170-pdf.pdf.

[46] Katz VS, Ang A, Suro R (2012). An Ecological Perspective on US Latinos’ Health Communication Behaviors, Access, and Outcomes. Hispanic Journal of Behavioral Sciences.

[47] Horowitz C, Brenner B, Lachapelle S, Amara D, Arniella G (2009). Effective Recruitment of Minority Populations through Community-Led Strategies. American Journal of Preventive Medicine.

[48] McCaffery K, Wardle J, Nadel M, Atkin W (2002). Socioeconomic Variation in Participation in Colorectal Cancer Screening. Journal of Medical Screening.

